# The Hybrid Mouse Diversity Panel: a resource for systems genetics analyses of metabolic and cardiovascular traits

**DOI:** 10.1194/jlr.R066944

**Published:** 2016-06

**Authors:** Aldons J. Lusis, Marcus M. Seldin, Hooman Allayee, Brian J. Bennett, Mete Civelek, Richard C. Davis, Eleazar Eskin, Charles R. Farber, Simon Hui, Margarete Mehrabian, Frode Norheim, Calvin Pan, Brian Parks, Christoph D. Rau, Desmond J. Smith, Thomas Vallim, Yibin Wang, Jessica Wang

**Affiliations:** Departments of Medicine,* David Geffen School of Medicine, University of California-Los Angeles, Los Angeles, CA; Microbiology,† David Geffen School of Medicine, University of California-Los Angeles, Los Angeles, CA; Human Genetics,§ David Geffen School of Medicine, University of California-Los Angeles, Los Angeles, CA; Molecular and Medical Pharmacology,§§§§ David Geffen School of Medicine, University of California-Los Angeles, Los Angeles, CA; Department of Preventive Medicine,** University of Southern California Keck School of Medicine, Los Angeles, CA; Department of Genetics,†† University of North Carolina, Chapel Hill, NC; Departments of Biomedical Engineering§§ University of Virginia, Charlottesville, VA; Public Health Sciences,††† University of Virginia, Charlottesville, VA; Departments of Computer Science,*** University of California-Los Angeles, Los Angeles, CA; Human Genetics,§§§ University of California-Los Angeles, Los Angeles, CA; Anesthesiology,†††† University of California-Los Angeles, Los Angeles, CA; Department of Nutritional Sciences,**** University of Wisconsin-Madison, Madison, WI

**Keywords:** aherosclerosis, osteoporosis, obesity, heart failure, microbiota, gene-by-diet interaction, gene expression, insulin resistance, gene mapping

## Abstract

The Hybrid Mouse Diversity Panel (HMDP) is a collection of approximately 100 well-characterized inbred strains of mice that can be used to analyze the genetic and environmental factors underlying complex traits. While not nearly as powerful for mapping genetic loci contributing to the traits as human genome-wide association studies, it has some important advantages. First, environmental factors can be controlled. Second, relevant tissues are accessible for global molecular phenotyping. Finally, because inbred strains are renewable, results from separate studies can be integrated. Thus far, the HMDP has been studied for traits relevant to obesity, diabetes, atherosclerosis, osteoporosis, heart failure, immune regulation, fatty liver disease, and host-gut microbiota interactions. High-throughput technologies have been used to examine the genomes, epigenomes, transcriptomes, proteomes, metabolomes, and microbiomes of the mice under various environmental conditions. All of the published data are available and can be readily used to formulate hypotheses about genes, pathways and interactions.

Common forms of cardiovascular and metabolic diseases are caused by the interactions of multiple genetic and environmental factors. The ability to interrogate the genomes of large numbers of individuals using high density genotyping and, more recently, next generation sequencing has enabled the identification of numerous loci robustly associated with many of the common disorders. However, efforts to extend these data to important biologic insights have progressed slowly. Human studies are often confounded by the difficulty of monitoring environmental factors and the inability to obtain relevant tissue samples for molecular analyses.

To address these issues, we have developed the Hybrid Mouse Diversity Panel (HMDP), a collection of approximately 100 inbred strains of mice exhibiting substantial diversity of most cardiovascular and metabolic traits relevant to human disease (1). The resource offers some important advantages for analysis of complex traits as compared with the traditional intercrosses between different mouse strains, including high-resolution association mapping and cumulative data. The HMDP strains have now been studied for a variety of metabolic and cardiovascular traits as well as various “omics” phenotypes (**Table 1**). The results have been collected in a database which can be searched and analyzed to identify novel disease genes, model biologic pathways, examine gene-by-environment, study host-gut microbiome relationships, and prioritize human genome-wide association study (GWAS) candidate genes.

**TABLE 1.** Clinical and molecular phenotypes studied in the HMDP resource

Trait	Diet
Plasma lipids	C, HF, ATH
Adiposity	C, HF, ATH
Osteoporosis	C
Blood cell levels	C, HF, ATH
IR	C, HF, ATH
Fatty liver disease	HF, ATH
Heart failure induced by isoproterenol	ISO
Atherosclerosis	ATH
Diabetic nephropathy	C
Transcript levels	
Liver	C, HF, ATH
Adipose	C, HF
Aorta	ATH
Hippocampus	C
Striatum	C
Skeletal muscle	HF
Heart	C, ISO
Protein levels, liver	C
Metabolites	
Liver	C
Plasma	HF, ATH
Gut microbiome	C, HF ATH
DNA methylation	C

Mice were maintained on chow (C), high-fat (HF), or atherogenic (ATH) diets or treated with ISO.

We anticipate that this review will primarily be of interest to cardiometabolic investigators interested in using data from the HMDP to help guide their research. Therefore, at the end of the review, in the Database section, we have discussed the kinds of questions that can be addressed using the data. Also, because many cardiometabolic researchers may not be versed in genetics approaches, we have defined some of the terms and concepts used in this review in **Table 2**.

**TABLE 2.** Glossary of genetics terms used in this review

Term	Definition
Biological scales	Various levels in the flow of information from DNA to proteins to metabolites to cell structures to cell interactions.
*Cis*-regulatory elements	Regions of DNA which regulate the transcription of genes, usually nearby, on the same DNA strand. Examples are promoters or enhancers.
Congenic strains	Strains in which a small region of the genome from one strain has been placed, by repeated crossing, onto the genetic background of a second strain.
Correlation	In statistics, a measure of the strength and direction of a linear relationship between two variables. Usually measured as a correlation coefficient.
eQTL	A genetic locus that controls the levels of a transcript.
GWAS	An examination of common genetic variation across the genome designed to identify associations with traits such as common diseases. Typically, several hundred thousand SNPs are interrogated using microarray technologies.
Haplotypes	Combinations of alleles at genetic loci that are inherited together.
Heritability	An estimate of the proportion of genetic variation in a population that is attributable to genetic variation among individuals.
Inbred strains	Strains in which a set of naturally occurring genetic variations have been fixed by many generations of inbreeding.
Linkage analysis	Analysis of the segregation patterns of alleles or loci in families or experimental crosses. Such analysis is commonly used to map genetic traits by testing whether a trait cosegregates with genetic markers whose chromosomal locations are known.
LD	In population genetics, LD is the nonrandom association of alleles. For example, alleles of SNPs that reside near one another on a chromosome often occur in nonrandom combinations owing to infrequent recombination. LD should not be confused with genetic linkage, which occurs when genetic loci or alleles are inherited jointly, usually because they reside on the same chromosome.
LD blocks	Regions of high correlation across genetic markers, which results from their linkage in *cis* on a chromosome and thus infrequent recombination during meiosis. LD blocks are often demarcated by recombination hot spots
Modules	In the context of network modeling, groups of components that are tightly connected or correlated across a set of conditions, perturbations or genetic backgrounds.
Natural genetic variation	Genetic variation that is present in all populations as a result of mutations that occur in the germline; the frequencies of such mutations in populations are affected by selection and by random drift. This is in contrast with experimental variation that is introduced by techniques such as gene targeting and chemical mutagenesis.
QTL	A genetic locus that influences complex and usually continuous traits, such as blood pressure or cholesterol levels.
RI strains	A set of inbred strains that is generally produced by crossing two parental inbred strains and then inbreeding random intercross progeny; they provide a permanent resource for examining the segregation of traits that differ between the parental strains.
Systems genetics	A global analysis of the molecular factors that underlie variability in physiological or clinical phenotypes across individuals in a population. It considers not only the underlying genetic variation but also intermediate phenotypes such as gene expression, protein levels and metabolite levels, in addition to gene-by-gene and gene-by-environment interactions.
*Trans*-regulatory factors	Factors which regulate the transcription of genes at a distance. Examples are transcription factors and microRNAs.

LD, linkage disequilibrium.

## THE HMDP

The HMDP was developed as a systems genetics resource similar to recombinant inbred (RI) strain sets (2, 3) or chromosome substitution strains (4), but with the added advantage of high-resolution association mapping (1). It consists of a set of 30 classic inbred strains chosen for diversity plus 70 or more RI strains derived primarily from strains C57BL/6J and DBA/2J (the BxD RI set) and A/J and C57BL/6J (the AxB and BxA RI sets). The classic strains provide mapping resolution, while the RI strains provide power. All of the chosen strains are commercially available from the Jackson Laboratory (https://www.jax.org) and all have been either sequenced (www.sanger.ac.uk/science/data/mouse-genomes-project) or densely genotyped (5).

### Cumulative data

In common with RI strains (6), the HMDP resource is renewable in the sense that the inbred strains are permanent. This allows multiple mice of the same genotype to be studied, increasing the accuracy of the data that are collected, and results derived from different studies of the HMDP can be integrated. For example, transcriptomic data obtained in one study (1) were used to interpret proteomic data (7) and metabolic data (8) obtained from a separate set of mice.

### High-resolution association mapping

**Fig. 1.**
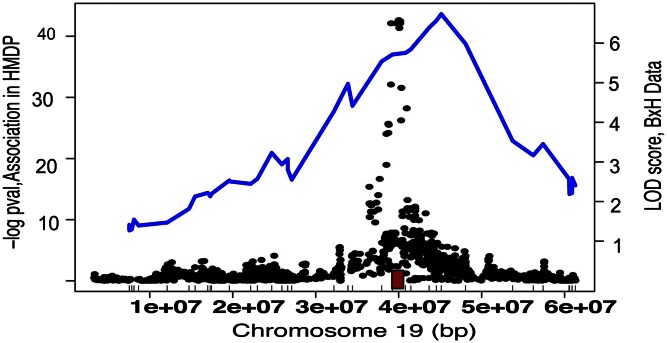
Greatly increased mapping resolution in the HMDP as compared with a traditional cross between two inbred strains. Shown is the mapping of a strong *cis*-eQTL, for the gene *Cyp2c37*, by linkage in an F2 cross (blue line) or by association in the HMDP (black dots). The position of the gene is indicated by the red box. The F2 cross included about 300 mice and global transcript levels were determined using microarrays. The figure is reprinted from (44), with permission.

The ability to perform high-resolution association mapping in the HMDP is based on the inclusion of about 30 “classic” inbred strains, which have undergone many generations of recombination since their origins from stocks of pet mice (9). This makes it possible to carry out association analysis much as in a human GWAS. Generally, it is possible to map complex traits to one to two megabase regions containing five to 20 genes or less using the HMDP, depending on the level of linkage disequilibrium and gene density of the region (1). This resolution is at least an order of magnitude improved as compared with traditional linkage analysis. For example, **Fig. 1** shows the mapping of a *cis*-expression quantitative trait locus (eQTL) in the HMDP and an F2 intercross. One important point to note is that because the classic inbred strains exhibit very significant population structure, it is essential that this is corrected to avoid false positive associations. This is conveniently accomplished using mixed model algorithms such as EMMA (10) or FaST-LMM (11). These algorithms essentially perform a *t*-test for association while correcting for population structure using a kinship matrix based on genotypes. Genome-wide significance is determined using simulation, a Bonferroni correction, or a false discovery rate (1, 12).

### Mapping power

With only 100 inbred strains in the HMDP, mapping power is considerably limited as compared with large intercrosses between pairs of inbred strains or human GWASs with thousands of samples. Nevertheless, simulation studies suggest that there is reasonable power to map loci that explain 5% or more of the trait variance (1). Because, as in humans, there are likely to be hundreds of loci that contribute to complex clinical traits, the mapping will generally detect only the handful of loci with strongest effects. Power can be increased by examining additional inbred and RI strains that have been genotyped (5, 13), but for practical reasons most studies have been limited to about 100 strains. Power can also be considerably increased while retaining high resolution by performing meta-analysis that incorporates data from traditional crosses (14, 15). Molecular phenotypes, such as transcript levels, protein levels, and metabolite levels, are generally determined by a much smaller number of loci than clinical traits and there is adequate power to map at least the major loci affecting these. For example, using expression arrays to quantitate liver transcript levels, about 2,500 significant *cis*-expression quantitative trait loci (eQTLs) were detected in liver (1), while about 5,000 *cis-*eQTLs were detected in cultured macrophages (16).

### Genetic diversity

The HMDP panel includes about 4,000,000 common SNPs, roughly similar to the number of common SNPs in human populations (17), and there is substantial variation of most clinical traits that have been examined, as discussed below. In contrast, the Collaborative Cross and the Diversity Outbred (18) include “wild-derived” strains, which increase the diversity by an order of magnitude (17). While there will certainly be greater total variation of most complex traits in the Collaborative Cross, there will also be greater genetic complexity, potentially complicating genetic dissection. Among the HMDP mice, about 40% of genes exhibit significant *cis*-eQTLs in various tissues, and the vast majority of genes exhibit secondary (*trans*-regulated) genetic variation.

### Relevance to complex human diseases

If the mouse is to serve as a model of common metabolic and cardiovascular traits, it is important that the relevant pathways be conserved in the two species. One measure of such conservation is the degree of overlap between mouse and human GWAS data. Studies in the HMDP for osteoporosis (19, 20), obesity (21), blood cell levels (22), and heart failure (23) suggest that the overlap will be substantial. We discuss an example of pathway conservation in the section on fatty liver disease.

## SYSTEMS GENETICS

The power of the HMDP for analysis of complex traits derives from the integration of genetics with global molecular phenotypes using “omics” technologies (Table 1). The natural variations found among the inbred strains of the HMDP directly perturb a substantial fraction of all genes, as judged by the number of genes exhibiting *cis*-eQTL or allele-specific expression (24, 25), and these, in turn, result in thousands of secondary perturbations. When the molecular and clinical traits are monitored together, relationships between them can be observed using mapping, correlation, and modeling [reviewed in (26)]. This is the basis of “systems genetics.”

### Genetic analysis of molecular phenotypes using high throughput technologies

Omics data can be analyzed using genetics in the same manner as other phenotypic traits. For example, variations in the levels of a transcript in a population can be treated as a quantitative trait and the genetic loci responsible can be mapped to regions of the genome using linkage or association analyses. Loci that reside near the genes whose transcripts are measured are likely to affect enhancer/promoter function and are thus often assumed to act in *cis*, while loci affecting expression of genes on other chromosomes or many megabases away on the same chromosome presumably act through diffusible factors and are thus assumed to act in *trans*. Such loci are termed eQTLs. Originally, individual transcript levels were quantitated in populations using hybridization or polymerase chain reaction amplification (27), but with the advent of expression arrays and RNA-Seq, it became possible to map eQTLs globally (1). Such studies have shown that genetic variations in gene expression are very common, affecting levels of thousands of genes in both human and mouse populations [reviewed in (26, 28)]. Moreover, it appears that a large fraction (∼85%) of the variations for common disease traits result from variations in gene expression rather than from structural (protein coding) variation [for example, (29)]. The levels of proteins and metabolites can also be quantitatively measured using high throughput technologies, and the loci controlling these can be similarly mapped to identify protein QTLs (pQTLs) or metabolite QTLs (7, 8).

### The flow of biologic information: from genes to molecular traits to clinical traits

**Fig. 2.**
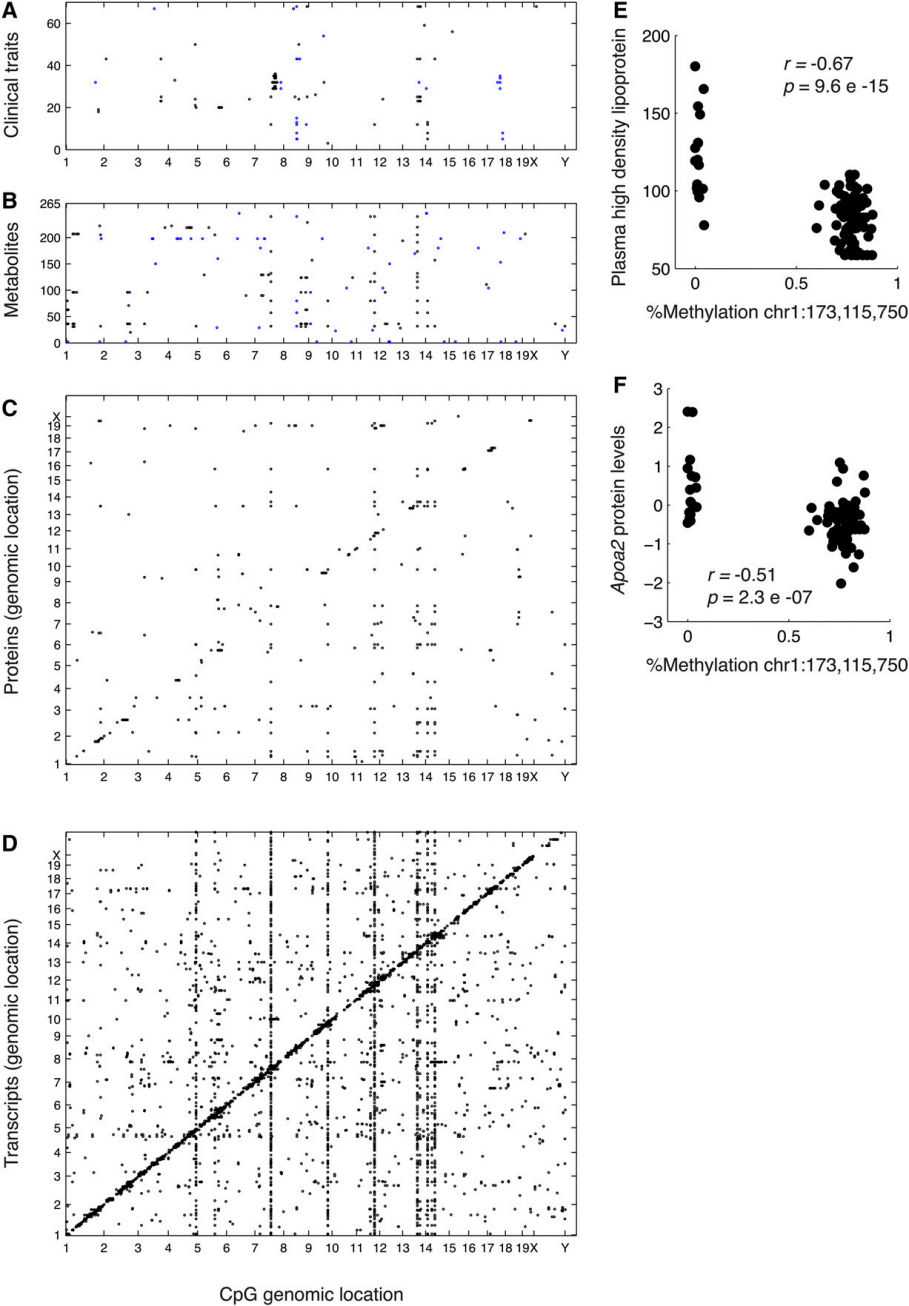
The flow of biologic information from liver DNA methylation to liver transcripts, proteins, and metabolites, and then clinical traits. The genomic positions of hypervariable CpGs are shown on the x axes and the y axes denote clinical traits (A), metabolites (B), proteins (C), or transcripts (D). In (C) and (D), the proteins or transcripts are plotted on the y axis according to the location of the encoding gene. Each dot is a significant association at the corresponding Bonferroni thresholds across CpGs tested with levels of clinical traits or levels of metabolites, proteins, or transcripts in liver. E, F: The association of percent methylation of a CpG on chromosome 1 at 173,115,750 base pairs (x axis) versus the levels of plasma HDL cholesterol (E) or apoAII (F). Reproduced from (63), with permission.

Whereas common disease traits are complex, influenced by tens or hundreds of loci, molecular traits tend to be much simpler. For example, *cis*-eQTLs often explain a large fraction of the variance of the transcript levels. A key aspect of the systems genetics approach is that molecular traits can thus constitute a bridge of sorts between DNA variation and clinical traits. An example of the application of such “vertical” omics is shown in **Fig. 2**. Several million sites of DNA methylation were identified in livers of the HMDP strains, using reduced representational bisulfite sequencing, and 22,000 sites that exhibited substantial genetic variation in methylation levels were selected. These were then tested for significant association with molecular traits, as quantitated by expression arrays, proteomics, and metabolomics, as well as clinical traits. The flow of biologic information is apparent at the “hotspot” loci where differences in DNA methylation at a single locus can be seen to influence the levels of multiple transcripts, proteins, and metabolites.

As illustrated below, omics data can be used to identify candidate genes for clinical traits using correlation and causality testing (30–32). Interactions between genes and their relationships to clinical traits can also be examined using enrichment analyses or network modeling (33, 34). Finally, subclinical phenotypes can provide an additional useful “bridge” between molecular phenotypes and the more complex clinical traits; for example, Attie and Kebede studied insulin secretion by isolated pancreatic β cells as a subphenotype for diabetes (35). In the sections below, we discuss the various datasets that have been generated and provide examples of the types of analyses that have been performed.

## TRAITS RELEVANT TO COMMON DISEASES

### Osteoporosis

Bone mineral density (BMD), a trait relevant to osteoporosis, is highly heritable in mice. Farber and colleagues examined variation of BMD among the HMDP strains and, using association and network modeling, have uncovered several novel genes, some of which also influence BMD in humans (19, 20). GWASs in the HMDP for total body, spinal, and femoral BMD revealed four significant associations (chromosomes 7, 11, 12, and 17) harboring between 14 and 112 genes each. This was reduced to 26 functional candidates by identifying those genes that were regulated by local eQTLs in bone or that harbored potentially functional nonsynonymous coding variants. A candidate at the strongest locus (chromosome 12) was a nonsynonymous SNP in the additional sex combs-like 2 (*Asxl2*) gene. The role of the gene was confirmed by showing that *Asxl2* knockout mice exhibit reduced BMD (19) and this has been confirmed in subsequent studies (36). It is noteworthy that the human *ASXL2* locus exhibits a suggestive association with BMD.

**Fig. 3.**
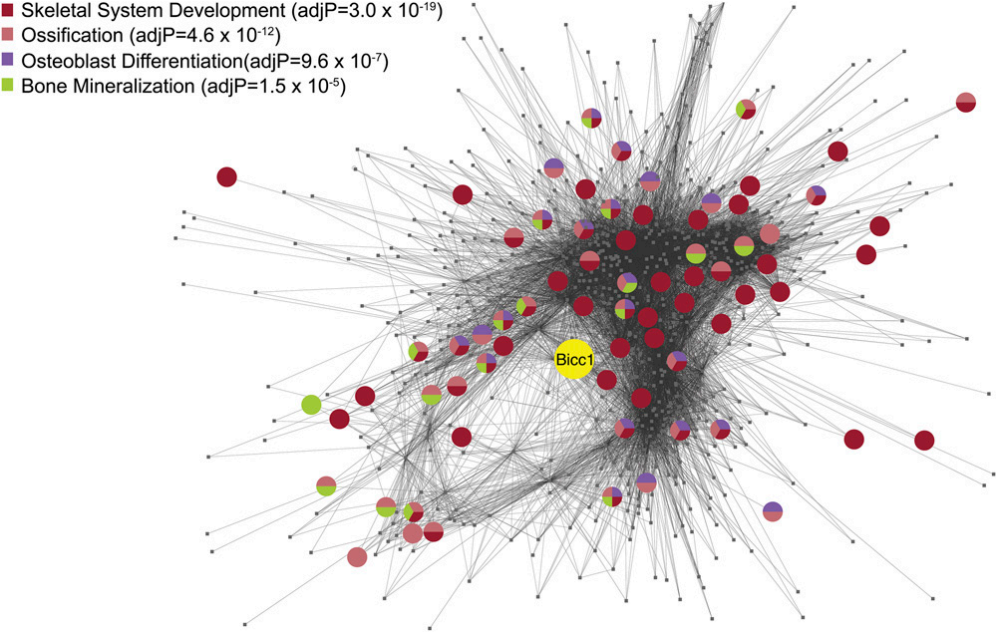
Network analysis predicts that *Bicc1* plays a role in osteoblast differentiation. *Bicc1* is a member of module 6 in a coexpression network based on global gene expression in bone tissue of the HMDP. The nodes represent genes and the lines indicate connections based on coexpression across the HMDP strains. The location of *Bicc1* is highlighted and each node is colored based on gene ontology annotations listed in the top left corner. Reproduced from (20), with permission.

To model biologic interactions of genes involved in BMD, the investigators used coexpression network analysis, an approach that partitions genes into modules, along with causality modeling (31, 37). A graphic representation of one such module enriched in BMD genes is shown in **Fig. 3**. Such network modeling studies suggested a function for *Asxl2* in osteoclast differentiation and this was validated by showing that knockdown of *Asxl2* in bone marrow macrophages impaired their ability to form macrophages. Two additional genes involved in osteoblast differentiation, *Maged 1* and *Pard6g*, were identified using analyses of a coexpression network module containing many genes that define the osteoblast lineage. Furthermore, the module was shown to be strongly regulated by the *Wnt* signaling agonist, *Sfrp1* (38). Recently, bone expression data from the HMDP were used to follow up on a BMD locus previously identified in a traditional F2 cross between strains C3H/HeJ and C57BL/6J. These studies revealed *Bicc1* as a novel determinant of osteoblastogenesis and BMD in both mice and humans (20).

### Obesity and dietary responsiveness

**Fig. 4.**
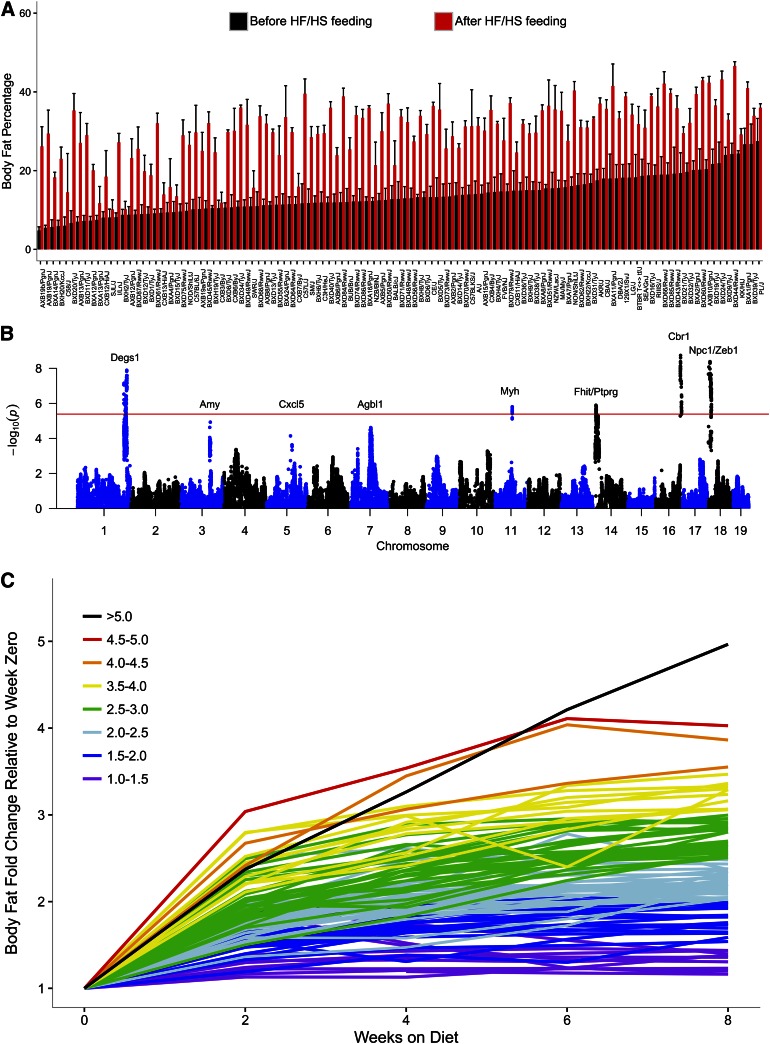
Genetic control of response to high-fat (HF) high-sucrose (HS) diet. Mice of the HMDP strains (six to eight male mice per group) were maintained on a low-fat chow diet until 8 weeks of age, when they were placed on a high-fat (32% kcal) and high-sucrose (25% kcal) diet for 8 weeks. The percent body fat on chow or on high-fat diet is shown in (A) and a GWAS of the percent body fat change following feeding of the diet is shown in (B). The red line in (B) indicates the threshold for genome-wide significance and likely candidate genes under each peak are indicated. The increase in percent body fat in response to the diet largely plateaus after about 4 weeks (C), consistent with a genetically controlled “setpoint” model of obesity (21). Reproduced from (21), with permission.

The analysis of obesity in humans is confounded by environmental factors such as the inability to monitor food intake. The HMDP has been particularly useful in examining the response to a high-fat dietary challenge because the same genetic backgrounds can be examined under different conditions. As shown in **Fig. 4A**, the HMDP strains exhibit substantial variation in body fat percentage on both chow and high-fat diets. The heritabilities for both fat as a percent of body weight as well as the response to a high-fat diet were in the range of 80%. Genome-wide association analyses of the HMDP identified eight significant/suggestive loci associated with obesity traits, such as body fat percent change in response to the diet (Fig. 4B), several of which overlapped with human GWAS loci for body mass index (21). For example, the chromosome 18 locus contains the endosomal/lysosomal Niemann-Pick C1 (*Npc1*) gene, a human GWAS hit (39, 40). A previous study with heterozygous knockout mice for *Npc1* revealed increased responsiveness to a high-fat diet as compared with wild-type mice, whereas there was no effect on a low-fat diet (41). This is precisely the phenotype observed in the HMDP: mice with reduced *Npc1* expression due to a *cis*-eQTL had increased adiposity on the high-fat diet, but not the chow diet. Other strong candidates are the amylase (*Amy*) genes on chromosome 3, which show copy number variation associated with altered expression levels, and *Degs1*, a fatty acid desaturase involved in the metabolism of bioactive sphingolipids. These same mice were examined for global transcript levels in liver, adipose, and muscle, as well as metabolites in plasma. A list of the most strongly correlated genes revealed many known to contribute to obesity, such as *Lep*, *Sfrp5*, *MIxipl*, *Dgat1*, and *Nnmt* (21).

These results have some important implications for the current “epidemic of obesity”. Thus, the findings support the concept of a genetically determined “setpoint,” because almost all of the strains studied reached a plateau level of body fat following the initial weight gain (Fig. 4C). The final plateau level was dependent on the genetic background between strains and was only weakly correlated with food consumption (21), although within a strain there was strong correlation between food intake and the development of obesity. Moreover, cross-fostering studies (in which the microbiomes of different strains are exchanged) showed that gut microbiotas are responsible, in part, for the differences in response to dietary challenge (42). This is consistent with the idea that subtle changes in microbiota composition may have contributed, in part, to the increased prevalence of obesity (43).

### Insulin resistance and type 2 diabetes

Insulin resistance (IR) is characterized by the failure of tissues to respond appropriately to insulin. It is strongly associated with obesity and contributes importantly to type 2 diabetes, fatty liver disease, and cardiovascular disease. Analysis of IR in humans is confounded by environmental factors, sex differences, age, and disease pathology and, despite large GWASs, there has been limited success in identifying the genetic factors and pathways contributing to IR. Analysis of the HMDP strains revealed large differences in IR when fed a diet rich in fat and refined carbohydrates along with striking sex differences. More than 15 genome-wide significant loci for traits associated with IR were identified and a novel IR gene, *Agpat5*, was validated. Mice in which *Agpat5* expression was suppressed, using an antisense oligonucleotide, had reduced plasma insulin levels and increased ability to clear glucose (12). *Agpat5* is a mitochondrial lipid acyltransferase involved in the conversion of lysophosphatidic acid to phosphatidic acid (12). Systems genetics analyses involving global transcript levels in liver and adipose tissue, as well as plasma metabolites, implicated a number of additional genes and revealed a significant correlation with plasma arginine levels (12).

### Fatty liver disease

Non-alcoholic fatty liver disease (NAFLD) encompasses a wide spectrum of liver abnormalities ranging from benign accumulation of lipids (steatosis) to inflammation and fibrosis (non-alcoholic steatohepatitis) to cirrhosis, and then end stage liver disease and cancer. As yet, human GWASs have succeeded in identifying only a handful of genes significantly associated with NAFLD and these explain a tiny fraction of disease heritability. NAFLD is strongly associated with obesity, diabetes, and dyslipidemia, and the “epidemic of obesity” has resulted in a high prevalence of NAFLD (20–30% of Western populations).

To identify genetic and environmental factors contributing to NAFLD, liver steatosis and related clinical and molecular traits were studied in the HMDP following feeding of a high-fat high-carbohydrate diet for 8 weeks (34). More than a 30-fold variation in liver TG was observed and, as in human populations, this was strongly associated with both body fat and IR, which together explained more than 40% of the variation in liver TG. GWASs revealed four loci significantly associated with hepatic TG levels, and candidates of each of the loci were screened using gene expression data (*cis*-eQTL, correlation with trait) and coding sequence variation, available in the Sanger database as discussed above. The *Gde1* gene in the chromosome 7 locus, containing a total of 17 genes, was selected on the basis of a strong *cis*-eQTL and strong correlation with hepatic TG content in both liver and adipose. Its role in steatosis was confirmed by showing that Gde1 overexpression and shRNA knockdown in liver using adenoviral delivery led to reciprocal effects in liver TG accumulation (44). *Gde1* encodes glycerophosphodiester phosphodiesterase 1, a broadly expressed integral membrane protein that catalyzes the degradation of deacylated phospholipids, such as glycerophosphoethanolamine and glycerophosphocholine. *Gde1* has no direct role in TG biosynthetic pathways; however, one of the end products of the phosphodiesterase reaction is glycerol 3-phosphate, the precursor for TG biosynthesis. In addition, *Gde1* may affect hepatic metabolic homeostasis through altering the availability of bioactive phospholipids and metabolites. How the variation in liver TG in the HMDP strains will correlate with subsequent pathologies is unknown, but liver TG levels were strongly associated with plasma alanine aminotransferase levels, a measure of liver injury. Prolonged feeding studies or stronger stressors will be required to examine the further progression of the disease.

NAFLD nicely illustrates the concordance of human and mouse disease pathways. At the present time, there is strong evidence from human studies for the involvement of six genes in susceptibility to NAFLD (**Table 3**). In the HMDP, five out of six of these genes exhibited significant correlation, in terms of gene expression in adipose or liver, with hepatic TG levels. Some of these associations (those with *cis*-eQTLs) may result from direct genetic variation driving the expression of these genes, whereas the others may be secondary.

**TABLE 3.** Concordance of human and mouse NAFLD GWAS genes

Gene	r	*P*	*cis-*eQTL	Tissue
*Gckr*	0.19	0.04	NS	Liver
*Ncan*	0.37	6 × 10^−5^	3 × 10^−8^	Adipose
*Tm6 sf2*	−0.23	0.01	NS	Adipose
*Lyplal1*	0.27	0.003	2 × 10^−30^	Liver
*Trib1*	0.24	0.012	NS	Adipose
*Pnpla3*	0.08	0.424	NS	Liver

Six genes, listed here, have been associated with NAFLD in human studies. Transcript levels for these genes were determined in livers and gonadal adipose tissue of the HMDP. Five of the six (the exception being *Pnpla3*) exhibited significant correlation (r) with hepatic TG levels in mice fed a high-fat high-carbohydrate diet in either liver or adipose. Two of the five had strong *cis*-eQTLs in liver (44).

### Heart failure

Heart failure is a very common cause of death, with a lifetime risk of more than one in nine in developed countries. Characterized by loss of cardiac output, heart failure is a heterogeneous disorder associated with complex pathological features, including contractile dysfunction, fibrosis, and hypertrophy. It is a highly heterogeneous disorder that results from many different chronic stressors, most notably hypertension and injury following myocardial infarction. The heterogeneity has complicated human GWASs and only a small number of significant loci have been identified despite meta-analyses of tens of thousands of patients (45, 46). To model heart failure in the mouse, Rau et al. (23) administered a β-adrenergic agonist, isoproterenol (ISO), to the HMDP for 3 weeks using an implanted pump. The strains showed considerable variability in the development of hypertrophy, fibrosis, and changes in heart function (based on echocardiography parameters). GWASs revealed 7 significant and 17 suggestive loci, containing an average of 14 genes in linkage disequilibrium with the peak SNP, for cardiac hypertrophy, fibrosis, and surrogate traits relevant to heart failure. A number of loci contained highly promising candidate genes, including genes known to contribute to Mendelian cardiomyopathies in humans or having established roles in cardiac pathology, as well as novel candidates based on systems genetics strategies.

**Fig. 5.**
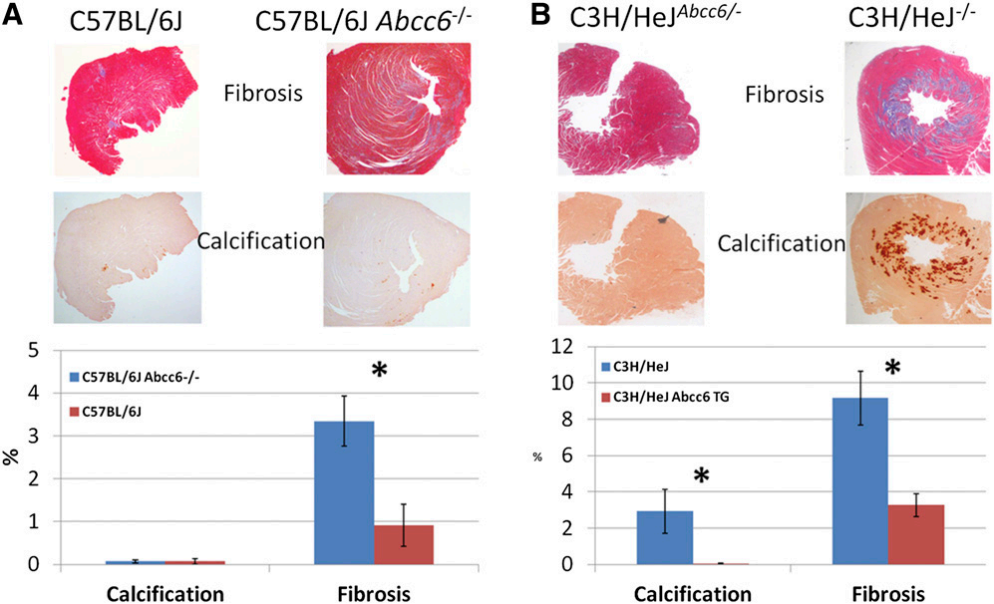
*Abcc6* deficiency contributes to cardiac fibrosis following treatment with the β-adrenergic agonist, isoproterenol. A: Shows either wild-type C57BL/6J mice or C57BL/6J mice homozygous for a null (gene targeted) allele of *Abcc6* (Abcc6^−/−^) following treatment with isoproterenol for 3 weeks. Neither strain developed significant calcification (stained with Alizarin Red) but the Abcc6^−/−^ developed substantially increased fibrosis (stained blue with Masson’s trichrome). B: C3H/HeJ mice are naturally deficient in *Abcc6* due to naturally occurring splicing mutation and when treated with isoproterenol develop extensive fibrosis and calcification in the heart. In contrast, C3H/HeJ mice carrying one copy of a genomic *Abcc6* clone as a transgene (C3H/HeJ Abcc6^−/−^) were resistant to both fibrosis and calcification. Reproduced from (23), with permission.

A strong candidate in a chromosome 7 locus for fibrosis was *Abcc6*, an orphan transporter that is the cause of the disorder, pseudoxanthoma elasticum, characterized by chronic calcification of a number of soft tissues, including heart. Mutations of the gene occur among a number of common mouse strains, such as DBA/2J and C3H/HeJ, where they cause calcification of heart and other tissues in older mice beginning at about 6 months of age (47). To test the role of *Abcc6* in ISO-induced fibrosis, gene-targeted mice on a C57BL/6J background were examined following ISO treatment. As compared with the wild-type mice the level of fibrosis (as measured by collagen content) in the knockout mice was substantially increased (**Fig. 5A**). Similarly, on a C3H/HeJ background, which carries a naturally occurring *Abcc6*-null mutation, mice expressing a genomic *Abcc6* transgene were rescued from fibrosis (22) (Fig. 5B).

### Plasma lipids

As compared with humans, mice have relatively low levels of LDLs and TG-rich lipoproteins and somewhat elevated levels of HDLs (48). Even when fed high-fat diets, the levels of LDL cholesterol and TGs remain relatively low. Higher levels of these, a prerequisite for the development of atherosclerotic lesions, can be achieved by feeding a diet containing cholic acid or introducing mutations in certain lipid transport proteins, such as the LDL receptor or apoE. Plasma lipid levels in the HMDP have been determined for mice maintained on chow (1) and high-fat (21) diets, as well as on a hyperlipidemic [*APOE*-Leiden, cholesteryl ester transfer protein (CETP) transgenic] background (49). The observed loci for lipid levels have generally been consistent with those identified in traditional crosses, but with greatly improved resolution (1). A meta-analysis of data from the HMDP, as well as several traditional crosses (a total of 4,965 mice), identified a total of 26 significant loci for HDL cholesterol levels (14).

### Atherosclerosis

**Fig. 6.**
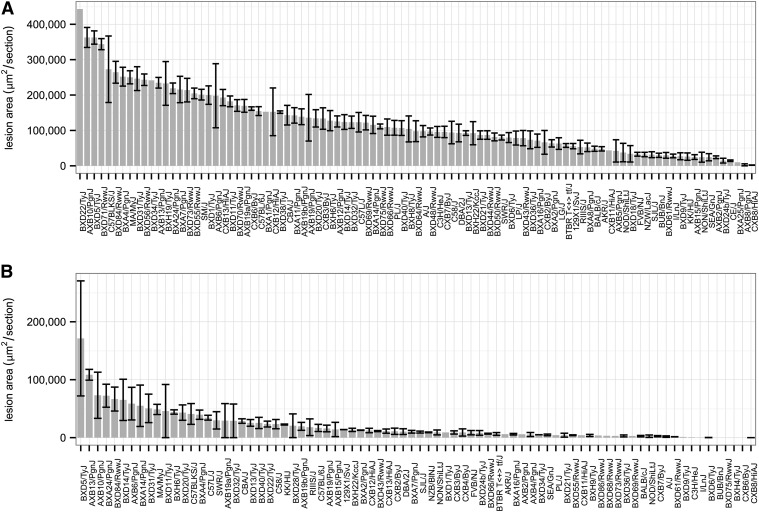
Atherosclerosis in the HMDP. Atherosclerosis lesion size (μm^2^ ± SEM) in the proximal aorta was quantitated in 697 female (A) and 281 male (B) mice using oil red O staining. In each panel, stains are arranged in rank order by strain-average lesion area. As discussed in the text, the null mice were on an APOE-Leiden, CETP transgene background and were fed a Western diet for 16 weeks. Data from Bennett et al. (49) with permission.

The mouse has become the most widely used animal model of atherosclerosis and there have been thousands of reports of candidate gene studies. As discussed above under the section on plasma lipids, most studies have been carried out on *Ldlr*^−/−^ or *Apoe*^−/−^ genetic backgrounds to raise the levels of atherogenic lipoproteins such that the mice develop significant lesions. The lesions share a number of characteristics with human lesions, and many human risk factors, such as hyperlipidemia, low HDL, hypertension, and inflammatory markers, replicate in mice. To examine atherosclerosis in the HMDP, Bennett et al. (49) used an F1 hybrid strategy in which the dominant acting atherosclerosis-promoting transgenes, human APOE-Leiden and human CETP, were bred from strain C57BL/6J onto over 100 different strains of the HMDP. Thus, the mice examined consisted of a genetic background derived from 50% C57BL/6J and 50% from the other strain. They were then fed a “Western” diet containing 1% cholesterol for 16 weeks and aortic lesion sizes were assessed. In addition, global gene expression was quantitated using arrays in the aorta and the liver, and levels of lipids, glucose, insulin, numerous cytokines, and a panel of metabolites were quantitated in the plasma. As shown in **Fig. 6**, despite the fact that all the mice consisted of 50% C57BL/6J background, there was well over a 600-fold range of variation in lesion sizes. While males tended to have lesion sizes several-fold smaller than females, the sizes of lesions in males and females were very significantly correlated (*r* = 0.474, *P* = 2.6 × 10^−15^). Because C57BL/6J mice have a roughly intermediate lesion size in both males and females, the very small lesions (less than half the size of those in C57BL/6J) cannot be explained by additive models of inheritance. The relationships between atherosclerosis and various risk factors in mice closely resembled those in humans (49). The data reported in the study provide a rich resource for further studies of atherosclerosis; for example, a number of relevant traits were mapped with high-resolution and a number of novel metabolite associations were observed. Furthermore, the expression data can be used to identify novel candidate genes or prioritize genes in human GWAS loci (29, 49).

A combination of human and HMDP expression data were used to model cross-tissue regulatory gene networks for atherosclerosis (50). Briefly, the authors constructed coexpression networks, identified modules associated with atherosclerosis, inferred causality using GWAS results, and, finally, identified what were termed “key driver” genes. The modeling was verified in part by comparing human and mouse networks and performing experiments with cell lines.

### Inflammatory responses

Many metabolic and cardiovascular traits have an important inflammatory component. To examine genetic contributions to inflammation, peritoneal macrophages from 92 strains of the HMDP were cultured and studied for genome-wide transcript levels before and after treatment with lipopolysaccharide (LPS) or oxidized lipids (Ox-PAPC) (16). A larger number of *cis*-eQTLs were identified in this study, as compared with in vivo tissues (5,217 in the control, 4,587 in the LPS, and 4,747 in the Ox-PAPC, as compared with 2,000–4,000 in most tissue studies). Presumably, this reflects reduced environmental effects and a more homogeneous cellular composition. Between 9,000 and 18,000 *trans*-eQTLs were also identified although, because of the problem of multiple comparisons, many of these are likely to be false positives (51). A number of the *trans*-acting loci were present as “hotspots,” particularly after LPS treatment. The largest such hotspot was on chromosome 9 at 119 Mb and included over 1,000 regulated genes, many of which were inflammatory cytokines or LPS-primary response genes. The locus contains 12 genes based on linkage disequilibrium of which only 6 were expressed in macrophages These were systematically tested using siRNA knockdown and the *trans* regulation of most of the genes was shown to be due to *2310061C15Rik*, a poorly characterized gene with homology to a mitochondrial protein involved in cytochrome C oxidase biogenesis (16). These data provide a rich resource for further studies of inflammatory interactions, including pathogen interactions; for example, periodontal bone loss in response to LPS varies strikingly in the HMDP (52).

### Type 1 diabetes and diabetic nephropathy

In some studies, only a fraction of the number of strains required for association mapping of traits have been characterized. One such study involves analysis of kidney disease in the context of type 1 diabetes (53). The authors bred the DBA/2J.*Akita* transgenic mouse model of type 1 diabetes to 28 of the HMDP strains and examined histologic and molecular parameters associated with diabetic nephropathy in diabetic mice and nondiabetic littermates. The most striking observed phenotype was urine albumin-to-creatinine ratios, which increased 2- to 6-fold over euglycemic control values for most strains, but more than 10-fold in six strains, including 50- and 83-fold in two strains, NOD/ShiLtJ and CBA/J, respectively (53).

### Other clinical traits

A variety of nonmetabolic traits are being studied in the HMDP. For example, the HMDP strains differ strikingly in hearing parameters and hearing loss due to noise. A number of loci were identified in association studies (15, 54) and *Nox3* was shown to be critical for noise-induced hearing loss (55).

Conditioned fear phenotypes and global transcript levels for hippocampus and striatum were determined in the HMDP strains (17). A total of 27 behavioral quantitative trait loci were mapped and these results were integrated with eQTL results. Coexpression networks were constructed for hippocampus and striatum, and modules strongly associated with fear traits were identified. Similarities and differences in modules in the two brain regions were examined (17).

## BASIC STUDIES

### Gene-by-environment interactions

**Fig. 7.**
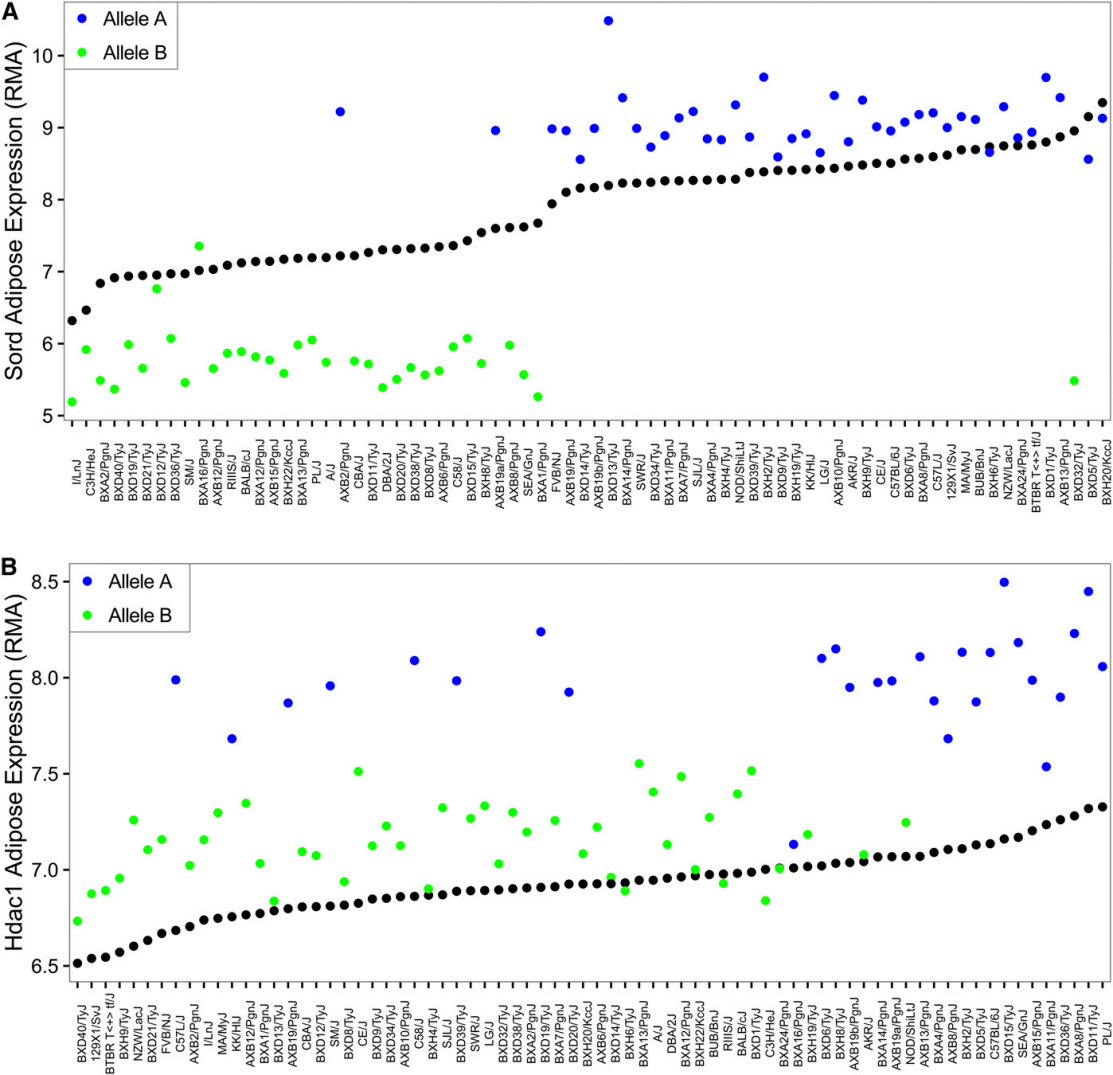
Gene-by-environment interactions in response to a high-fat high-sucrose (HF/HS) diet. Shown are adipose transcript levels for two genes, sorbitol dehydrogenase (A) and histone deacetylase 1 (B), in mice fed either the chow diet (black dots) or the HF/HS diet (colored dots). The strains are rank ordered by transcript levels on the chow diet and the transcript levels on the HF/HS diet are colored according to the genotype of the peak *cis*-eQTL. In the case of sorbitol dehydrogenase, gene expression levels in mice with allele B are repressed by the diet, whereas those with allele A are induced. In the case of histone deacetylase 1, the induction is much larger in mice with genotype A than genotype B.

While human GWASs have identified many loci for metabolic and cardiovascular traits, a major limitation is the inability to examine environmental interactions. When the HMDP mice were challenged with various environmental conditions, a high-fat/high-sucrose diet (12, 21), a high-fat/high-cholesterol diet (49), or isoproterenol treatment (23), virtually all clinical traits examined and hundreds of molecular traits, such as transcript levels, showed evidence of gene-by-environment (GxE) interactions (for example, see **Fig. 7**). Most striking were inflammatory responses of peritoneal macrophage to bacterial LPS, where a number of hotspots affecting the responses of hundreds of genes were identified (16). Because the majority of common genetic variation is regulatory rather than protein coding (56), it is not surprising that GxE interactions occur so frequently. It is likely that changes in transcription factor binding related to sequence variation will be a major mechanism driving *cis*-regulated GxE interactions such as those in Fig. 7, although any of the events that are critical for gene expression could be involved, including chromatin interactions, chromatin state, alternative splicing, and posttranslational modifications. Many of the *trans*-regulated effects could result from genetic differences affecting the metabolism of dietary components or drugs. The gut microbiome, for example, is likely to be an important mediator of environmental responses, as discussed in the section below.

### Gene-by-gene interactions

The importance of gene-by-gene (GxG) interactions in common disease in humans has been controversial, but studies in mice strongly point to their importance (57, 58). The significance of GxG interactions can be examined globally by comparing “broad sense” heritability (the sum of all genetic influences) with “narrow sense” heritability (the portion due to additive effects and not including GxG interactions). For example, a study of numerous traits in haploid yeast suggested that broad sense was substantially larger than narrow sense heritability for some traits but not others (59). Whereas such parameters are difficult to estimate in humans, they can be studied more accurately in mice because genetically identical replicates (members of inbred strains) are available and the environment can be controlled. Indeed, using the HMDP, traits such as heart failure and atherosclerosis appear to have considerably greater broad sense than narrow sense heritability (49).

### Epigenetics

High-resolution genome scale epigenetic profiling using next generation sequencing (ChIP-Seq, DNase-Seq, FAIRE-Seq, bisulfite sequencing, etc.) has enabled analysis of the regulatory variation in which genetic variants are likely to act (60, 61). A variety of epigenetic marks in liver have been examined in a subset of the HMDP (62) and DNA methylation has been examined in 90 HMDP strains (63, 64). Much of the epigenetic variation was found to be controlled in *cis* and was strongly associated with the expression levels of nearby genes, which were, in turn, associated with protein, metabolite, and clinical traits (see Fig. 2 for example). Figure 2 shows an example of a DNA methylation that occurs near the *Apoa2* gene on chromosome 1. The degree of methylation is strongly associated with the levels of apoA2 protein and HDL cholesterol (apoA2 is the second-most abundant protein in HDL). In addition to *cis* regulation, some instances of *trans* regulation were validated. For example, variable methylation of a cytosine-phosphate-guanine (CpG) on chromosome 13 was associated with the degree of methylation at hundreds of sites throughout the genome, as well as the expression of many genes. A strong candidate for mediating the effect was the nearby *Mtrr* gene, encoding methionine synthase reductase. The enzyme is part of the folate cycle, involved in the generation of methyl donors for DNA and histone methylation. To experimentally validate *Mtrr* as the causal gene, gene-trapped *Mtrr* mice with reduced gene expression were studied and found to affect a highly overlapping set of methylation sites (63).

The most striking finding from these studies was the strong association between certain variations in DNA methylation and complex clinical traits, such as HDL levels, IR, obesity, and blood cell levels. For example, Fig. 2E, F shows the association of a methylation site on chromosome 1 with HDL cholesterol levels and expression levels of the nearby apoA-II gene (*Apoa2*). For many complex traits, the associations with methylation were much stronger than with any nearby SNPs. Whether such strong associations result from effects on the expression of nearby genes or some other mechanism is unclear (64). Moreover, combinations of multiple methylation sites, identified using linear regression modeling, were capable of predicting complex phenotypes, such as BMD and blood cell traits. Notably, many of the loci containing these methylation sites did not overlap significantly with the SNP-based association (64).

### Genetic control of protein abundance

Mapping protein levels as a quantitative trait (pQTL) is a critical aspect of understanding regulatory variation in the context of common disease. Recent advances in mass spectrometry-based proteomic methods have now enabled quantitation of thousands of proteins. One important question is the relationship between transcript levels and protein levels as a function of genetic variation. Whereas transcript-protein correlations are clearly very strong between different cell types, the perturbations introduced by common genetic variation are much more subtle. This issue was evaluated in liver using the HMDP (7). Ghazalpour et al. (7) quantified over 5,000 peptides in the HMDP using a liquid chromatography-mass spectrometry reference-based labeling approach. Based on this, a set of 485 most reliable proteins were selected and compared with levels of the corresponding transcripts. Although, in some cases, the correspondence was excellent and many highly significant pQTLs were mapped, about half of the protein-transcript pairs exhibited little or no correlation, even among the most heritable variations in transcript levels. A somewhat stronger correspondence was observed in yeast intercross population using green fluorescent protein tags to quantify single-cell protein abundance (59). Although technical factors undoubtedly contributed to the lack of correspondence, there are a number of ways in which protein levels might be regulated independently of transcript levels, including regulation of translation, codon constraint, RNA editing, alternative splicing, posttranslational modifications, and protein turnover. One particularly significant mechanism may involve protein complexes; thus, proteins which form complexes with other proteins likely have a specified stoichiometry, and if one protein is produced in excess of the other, it will likely undergo rapid degradation. In the study of Ghazalpour et al. (7), it is noteworthy that in the case of ribosomal proteins, many of which were detected, there was essentially no correspondence between transcript and protein levels. Presumably, any such proteins produced in excess of the levels that could be incorporated into ribosomes would be rapidly degraded.

### Regulation of metabolism

Recent advances in mass spectrometry and nuclear magnetic resonance have made high throughput analyses of hundreds of metabolites in biologic samples possible, and investigators have begun to utilize the relationships between metabolite levels and disease traits for use as biomarkers or elucidation of disease mechanisms. Human population studies of plasma metabolites have identified a number of disease associations and shown that levels of many metabolites are highly heritable (65). The HMDP offers an opportunity to integrate metabolite levels with epigenetic, transcriptomic, protein, and clinical data under controlled conditions (see Fig. 2) and studies of metabolite levels have been performed for liver and plasma when mice were fed either chow or high-fat diets (8, 11, 49). A number of conclusions emerged; for example, trimethylamine-*N*-oxide (TMAO) levels were found to be a strong predictor of atherosclerosis (49), as they are in humans. GWAS analyses resulted in the identification of numerous metabolite QTLs (mTQLs), and the causal genes for some of these differences were experimentally validated (8). In a study of liver metabolites in mice fed a chow diet, 40% of metabolites measured showed evidence for genetic regulation. In total, the 110 measured metabolites were found to be mapping significantly to 240 loci, and 36 metabolites were found to be significantly associated with clinical traits (8). This work also highlighted the value of using the HMDP to identify and validate candidate genes regulating metabolite levels by integrating the transcript eQTLs with the metabolite QTLs. Following this recipe, the authors were able to identify the causal genes affecting *N*-acetylglutamate and glycerol-3-phosphate levels in liver.

### Host-gut microbiota interactions

There is now overwhelming evidence that gut microbes can contribute to metabolic and cardiovascular disorders (66). A striking example is the association between levels of TMAO, a substance derived exclusively through the action of gut microbiota and cardiovascular disease. As yet, however, which microbes contribute to disease traits and what factors determine the composition of gut microbiota are poorly understood. Genetics provides a potentially powerful approach to address such questions, and to that end, Parks, Org, and colleagues (21, 42) profiled gut microbiota using 16S rRNA gene sequencing from over 100 HMDP strains. Remarkably, they observed very high heritability of microbiota composition, in the range of 0.5 for most genera (42). They also observed a number of relationships between gut microbiota composition and clinical traits. For example, a strong association between levels of *Akkermansia mucinophila*, a common microbe that resides in and digests the mucin layer of the intestine, and IR was observed (21). This was then tested experimentally by introducing the microbe into mice using gavage and, indeed, profound effects on IR and other metabolic traits were observed (42). In other studies, the composition of the gut microbiota was shown to contribute to differences in TMAO levels between inbred strains of mice (67, 68). Finally, cross-fostering studies, in which newborn mice are raised by foster mothers and consequently “inherit” their microbiota, suggested that differences in response to diet in the HMDP strains was due, in part, to the composition of the gut microbiota (42). Large human population studies of gut microbiota composition have been reported (69) and others are underway but, given the very large impact of diet and other environmental factors on gut microbiota, it will be challenging to tease out disease associations. The HMDP data constitute a powerful resource for further dissection of mechanistic host-gut microbiota interactions, enabling the formation of hypotheses that can then be examined in human studies.

### Sex differences

**Fig. 8.**
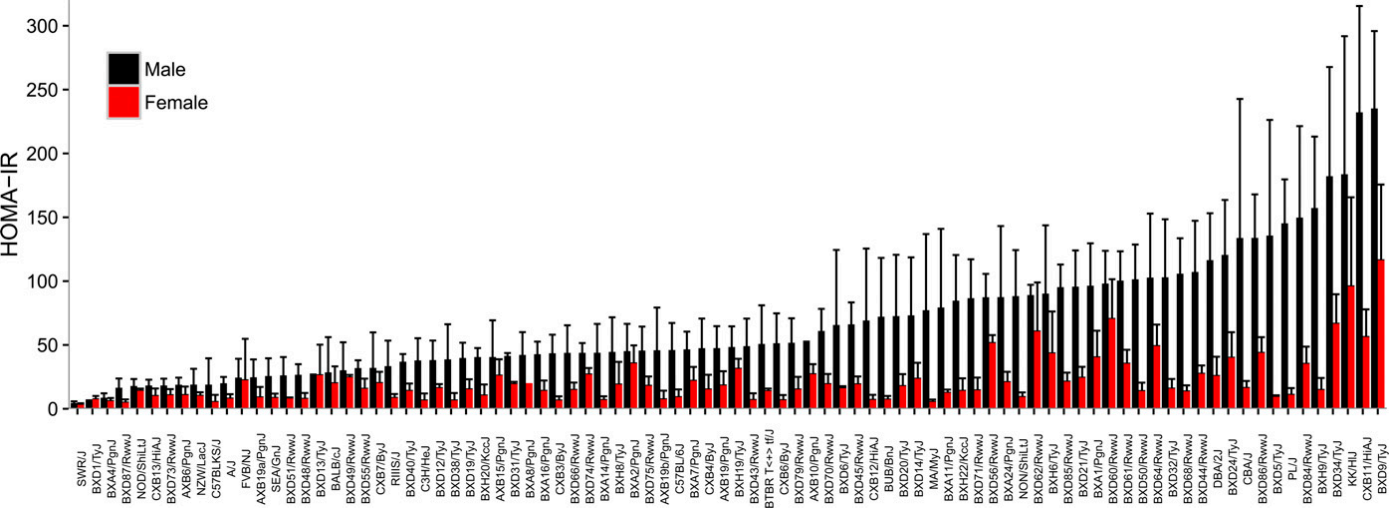
Sex differences in IR in the HMDP. HOMA-IR, a measure of IR based on glucose and insulin levels, was determined in the HMDP for males and females. In addition to large differences between strains, females clearly tended to be less insulin resistant than males. Reproduced from (12), with permission.

Most common diseases, including metabolic and cardiovascular diseases, differ in prevalence between men and women (70). In mice, such differences can be examined in detail, and previous studies have revealed thousands of differences in gene expression between sexes (71), most of them resulting from hormonal effects (72). In the HMDP, most clinical traits exhibited striking differences between males and females. For example, **Fig. 8** shows IR, quantitated as homeostatic model assessment (HOMA)-IR. While there is considerable genetic variation, it is clear that in the majority of strains, HOMA-IR is greater in males (12). While explanations for most of these differences are unknown, systems genetics approaches in the HMDP should be informative. For example, whereas in humans, males are more susceptible to atherosclerosis than females, the reverse is true in mice. Studies of a subset of HMDP mice revealed that levels of TMAO [a strong contributor to atherosclerosis, in humans and mice (49)] were much higher in females than in males, and analysis of hepatic transcript levels showed that this was due largely to greatly decreased levels of the enzyme, FMO3, in male mice due to repression by testosterone (67). In contrast, in humans, FMO3 expression is similar in males and females.

### Blood cell levels

The levels of the major blood cell groups, red cells, lymphocytes, monocytes, and granulocytes, vary considerably among the HMDP strains (22). A number of loci for each cell type were identified by GWASs, several of which overlap with loci observed in human studies. For example, five red cell trait loci were identified in the HMDP and four of these correspond to red cell loci reported in a recent human GWAS (73). A major locus affecting mean corpuscular volume and several other red cell traits mapped to *Hbb-b1*, a likely causal gene that is part of the β-globin cluster on chromosome 7 (22).

### Stem cells

Genetic factors controlling stem cell number, proliferation, and differentiation are poorly understood. Zhou et al. (74) utilized a GWAS approach in the HMDP to examine quantitative variations affecting levels of hematopoietic stem progenitor cells (HSPCs). They obtained bone marrow mononuclear cells from 12-week-old male HMDP mice and examined the frequency of various HSPC populations using flow cytometric analysis of lineage-specific cell surface markers. The markers included lineage [Liu] Sca-1^+^c-Kit^+^ [LSK], the more immature LSKCD150^−^CD48^−^ multipotent progenitors, and the most primitive LSKCD150^+^CD48^−^ cells. The frequencies of these varied approximately 120- to 300-fold across the 108 HMDP strains surveyed. This variation was largely genetic, with heritabilities ranging from 0.7 to 0.9. The three types of primitive HSPCs were correlated with each other and, the LSK and LSKCD150^−^CD48^−^ were modestly correlated with total white cell counts and the numbers of lymphocytes and monocytes. GWAS analyses identified multiple significant loci, several containing strong candidates for each of the HSPC levels. *Hopx*, located in a chromosome 5 locus Associated with LSKCD150^-^CD48^−^ cells, was selected for further analysis based on correlation of its expression with HSPC levels and a strong *cis*-eQTL. Its role was validated using knockout mice, which had decreased levels of LSKCD150^−^CD48^−^ cells, but no differences in LSK or LSKCD150^+^CD48^−^ cells (74).

## HMDP DATABASE AND ITS USE FOR CARDIOMETABOLIC RESEARCH

The data discussed above are organized on a server at UCLA and published data are available upon request from the corresponding author. Some of the data are also available through the Jax Phenome Database (phenome.jax.org) as well as the GeneNetwork database (www.genenetwork.org). Also, precomputed data, including trait-genome associations (for clinical and molecular traits), trait correlations, and expression data across tissues, can be easily searched at the Systems Genetics Resource (https://systems.genetics.ucla.edu/) (75). Below, we briefly outline how the database can be interrogated to address certain questions. The basic operations used are correlation, genetic mapping, and statistical modeling (26)

### What information can be gained about your gene of interest?

**Fig. 9.**
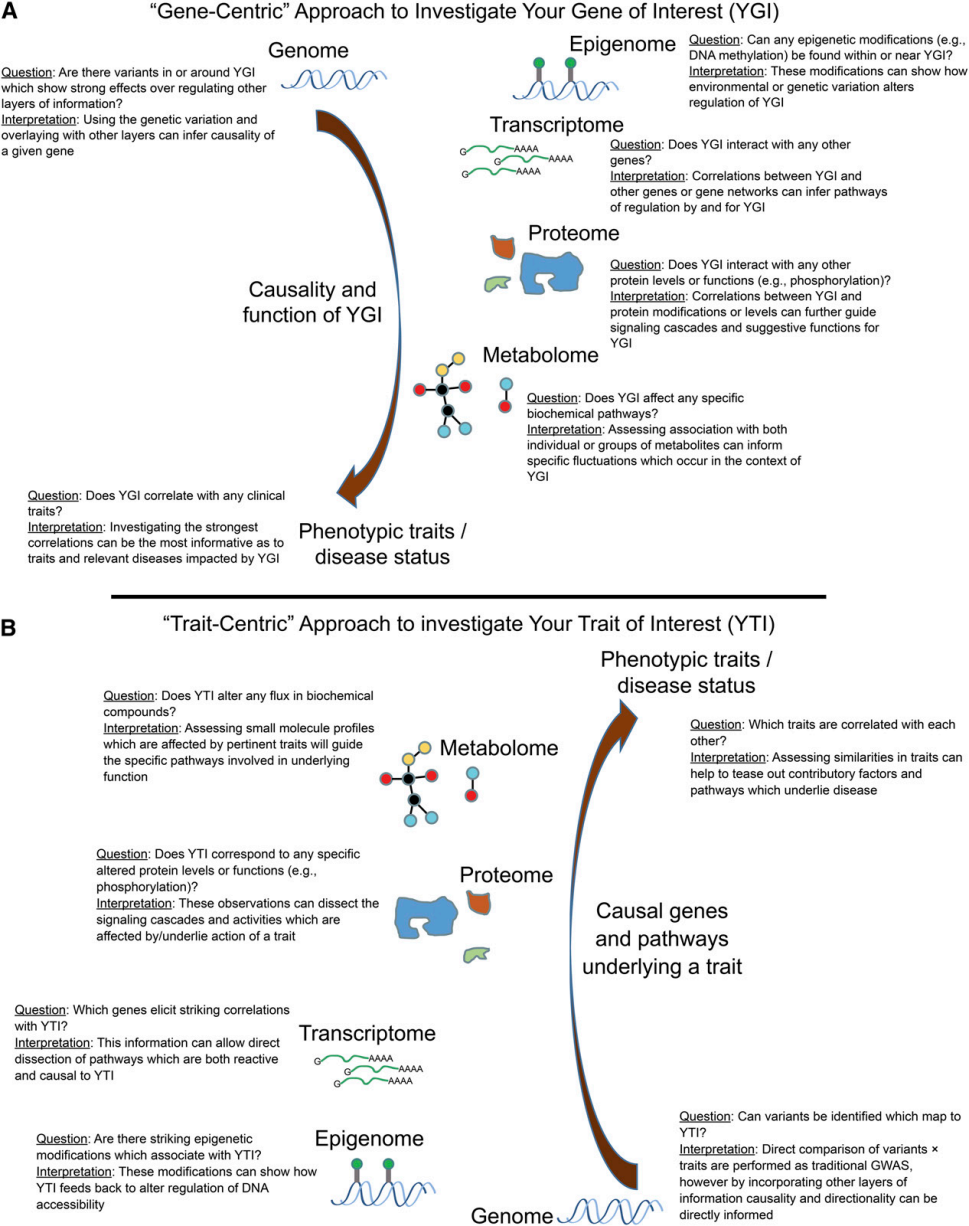
Application of the HMDP database to investigate genes or traits of interest. Hypothetical examples of how information from the HMDP can be utilized to explore relationships between genes (A) and traits (B) of interest and their relationships with multiple layers of information. For each layer, correlation analysis can be used to ask a specific question and interpret results which could elucidate novel functions and/or relationships of genes or traits of interest.

One informative operation is to obtain the list of clinical traits or molecular traits (other genes, proteins, metabolites) that are correlated with any gene of interest (**Fig. 9A**). There are several possible explanations for the correlation: Your gene of interest (YGI) may influence the other traits (causal, indicated by a red arrow), it may be perturbed by the other traits (reactive), or the correlation may result from the fact that both YGI and the correlated traits are regulated by some other factor, possibly another gene or a technical issue such as a batch effect. Such a list provides candidates for further study and can be broadly examined for pathway enrichment [for example, see (44)] thereby illuminating possible functions of YGI. It is also possible to perform causal modeling to help identify mechanistic interactions (30, 31). For example, if the expression of YGI is regulated by a strong *cis*-eQTL, one can ask whether other traits map to that same locus.

### What can I learn about my complex clinical trait of interest?

Similar to the analysis of YGI above, a useful operation is to examine the genes, proteins, or metabolites correlated with the clinical trait (Fig. 9B). The relationships may be causal, reactive, or independent, as discussed above. Also, one can map the major loci contributing to the traits of interest and subsequently prioritize the candidate genes at the loci using gene expression and sequence data. Finally, various kinds of modeling can be applied to identify sets of genes involved in the trait; for example, coexpression modeling can identify gene modules that can be tested for relationship to the trait using principal component analysis (76).

There are many other types of questions that can be addressed using the HMDP database. Examples include: What is the relationship between chromatin marks, gene expression, and clinical traits? What is the nature of gene-by-environment interactions? How does the host contribute to gut microbiota composition? What pathways are shared among disease traits? The approaches to these questions are discussed in the works reviewed above.

## CONCLUSIONS

The HMDP resource provides a means of formulating hypotheses about the interactions underlying complex metabolic and cardiovascular traits. Whereas QTL mapping using traditional crosses in mice succeeded in identifying numerous highly replicable loci, the poor resolution of linkage analysis, often tens of megabases, made the identification of strong candidates difficult. Consequently, only a modest number of causal genes were identified over the past twenty-five years (77). In contrast, since its development in 2010, studies by a small number of laboratories using the HMDP have validated well over a dozen novel genes underlying complex traits. Key to this has been the integration of high-resolution association mapping along with systems genetics analysis using high throughput data.

A large body of data has now been collected and is freely available to interested researchers. This includes hundreds of genome-wide significant loci, most containing less than a dozen genes, along with expression, proteomic, and metabolomics data to narrow the list of likely candidates. Apart from mapping, the lists of genes correlated with clinical traits contain many of the genes known to contribute to the traits [for example (21)] and is undoubtedly highly enriched for genes yet to be discovered. The resource also presents opportunities to examine fundamental issues such as GxE and GxG interactions, sex differences, and host-gut microbiota interactions.

## References

[1] Bennett B. J., Farber C. R., Orozco L., Kang H. M., Ghazalpour A., Siemers N., Neubauer M., Neuhaus I., Yordanova R., Guan B., 2010 A high-resolution association mapping panel for the dissection of complex traits in mice. Genome Res. 20: 281–290. 20054062 10.1101/gr.099234.109 PMC2813484

[2] Andreux P. A., Williams E. G., Koutnikova H., Houtkooper R. H., Champy M. F., Henry H., Schoonjans K., Williams R. W., and Auwerx J. 2012 Systems genetics of metabolism: the use of the BXD murine reference panel for multiscalar integration of traits. Cell. 150: 1287–1299. 22939713 10.1016/j.cell.2012.08.012 PMC3604687

[3] Williams E. G., and Auwerx J. 2015 The convergence of systems and reductionist approaches in complex trait analysis. Cell. 162: 23–32. 26140590 10.1016/j.cell.2015.06.024 PMC4493761

[4] Nadeau J. H., Forejt J., Takada T., and Shiroishi T. 2012 Chromosome substitution strains: gene discovery, functional analysis, and systems studies. Mamm. Genome. 23: 693–705. 22961226 10.1007/s00335-012-9426-y PMC3917716

[5] Rau C. D., Parks B., Wang Y., Eskin E., Simecek P., Churchill G. A., and Lusis A. J. 2015 High-density genotypes of inbred mouse strains: improved power and precision of association mapping. G3 (Bethesda). 5: 2021–2026. 26224782 10.1534/g3.115.020784 PMC4592984

[6] Toth L. A., Trammell R. A., and Williams R. W. 2014 Mapping complex traits using families of recombinant inbred strains: an overview and example of mapping susceptibility to Candida albicans induced illness phenotypes. Pathog. Dis. 71: 234–248. 24535895 10.1111/2049-632X.12160

[7] Ghazalpour A., Bennett B., Petyuk V. A., Orozco L., Hagopian R., Mungrue I. N., Farber C. R., Sinsheimer J., Kang H. M., Furlotte N., 2011 Comparative analysis of proteome and transcriptome variation in mouse. PLoS Genet. 7: e1001393. 21695224 10.1371/journal.pgen.1001393 PMC3111477

[8] Ghazalpour A., Bennett B. J., Shih D., Che N., Orozco L., Pan C., Hagopian R., He A., Kayne P., Yang W. P., 2014 Genetic regulation of mouse liver metabolite levels. Mol. Syst. Biol. 10: 730. 24860088 10.15252/msb.20135004 PMC4188043

[9] Silver L. M. 1995. Mouse Genetics: Concepts and Applications. Oxford University Press, Oxford, UK.

[10] Kang H. M., Zaitlen N. A., Wade C. M., Kirby A., Heckerman D., Daly M. J., and Eskin E. 2008 Efficient control of population structure in model organism association mapping. Genetics. 178: 1709–1723. 18385116 10.1534/genetics.107.080101 PMC2278096

[11] Lippert C., Listgarten J., Liu Y., Kadie C. M., Davidson R. I., and Heckerman D. 2011 FaST linear mixed models for genome-wide association studies. Nat. Methods. 8: 833–835. 21892150 10.1038/nmeth.1681

[12] Parks B. W., Sallam T., Mehrabian M., Psychogios N., Hui S. T., Norheim F., Castellani L. W., Rau C. D., Pan C., Phun J., 2015 Genetic architecture of insulin resistance in the mouse. Cell Metab. 21: 334–346. 25651185 10.1016/j.cmet.2015.01.002 PMC4349439

[13] Furlotte N. A., Kang E. Y., Van Nas A., Farber C. R., Lusis A. J., and Eskin E. 2012 Increasing association mapping power and resolution in mouse genetic studies through the use of meta-analysis for structured populations. Genetics. 191: 959–967. 22505625 10.1534/genetics.112.140277 PMC3389987

[14] Kang E. Y., Han B., Furlotte N., Joo J. W., Shih D., Davis R. C., Lusis A. J., and Eskin E. 2014 Meta-analysis identifies gene-by-environment interactions as demonstrated in a study of 4,965 mice. PLoS Genet. 10: e1004022. 24415945 10.1371/journal.pgen.1004022 PMC3886926

[15] Ohmen J., Kang E. Y., Li X., Joo J. W., Hormozdiari F., Zheng Q. Y., Davis R. C., Lusis A. J., Eskin E., and Friedman R. A. 2014 Genome-wide association study for age-related hearing loss (AHL) in the mouse: a meta-analysis. J. Assoc. Res. Otolaryngol. 15: 335–352. 24570207 10.1007/s10162-014-0443-2 PMC4010595

[16] Orozco L. D., Bennett B. J., Farber C. R., Ghazalpour A., Pan C., Che N., Wen P., Qi H. X., Mutukulu A., Siemers N., 2012 Unraveling inflammatory responses using systems genetics and gene-environment interactions in macrophages. Cell. 151: 658–670. 23101632 10.1016/j.cell.2012.08.043 PMC3513387

[17] Park C. C., Gale G. D., de Jong S., Ghazalpour A., Bennett B. J., Farber C. R., Langfelder P., Lin A., Khan A. H., Eskin E., 2011 Gene networks associated with conditional fear in mice identified using a systems genetics approach. BMC Syst. Biol. 5: 43. 21410935 10.1186/1752-0509-5-43 PMC3070648

[18] Iraqi F. A., Athamni H., Dorman A., Salymah Y., Tomlinson I., Nashif A., Shusterman A., Weiss E., Houri-Haddad Y., Mott R., 2014 Heritability and coefficient of genetic variation analyses of phenotypic traits provide strong basis for high-resolution QTL mapping in the Collaborative Cross mouse genetic reference population. Mamm. Genome. 25: 109–119. 24445421 10.1007/s00335-014-9503-5

[19] Farber C. R., Bennett B. J., Orozco L., Zou W., Lira A., Kostem E., Kang H. M., Furlotte N., Berberyan A., Ghazalpour A., 2011 Mouse genome-wide association and systems genetics identify Asxl2 as a regulator of bone mineral density and osteoclastogenesis. PLoS Genet. 7: e1002038. 21490954 10.1371/journal.pgen.1002038 PMC3072371

[20] Mesner L. D., Ray B., Hsu Y. H., Manichaikul A., Lum E., Bryda E. C., Rich S. S., Rosen C. J., Criqui M. H., Allison M., 2014 Bicc1 is a genetic determinant of osteoblastogenesis and bone mineral density. J. Clin. Invest. 124: 2736–2749. 24789909 10.1172/JCI73072 PMC4038574

[21] Parks B. W., Nam E., Org E., Kostem E., Norheim F., Hui S. T., Pan C., Civelek M., Rau C. D., Bennett B. J., 2013 Genetic control of obesity and gut microbiota composition in response to high-fat, high-sucrose diet in mice. Cell Metab. 17: 141–152. 23312289 10.1016/j.cmet.2012.12.007 PMC3545283

[22] Davis R. C., van Nas A., Bennett B., Orozco L., Pan C., Rau C. D., Eskin E., and Lusis A. J. 2013 Genome-wide association mapping of blood cell traits in mice. Mamm. Genome. 24: 105–118. 23417284 10.1007/s00335-013-9448-0 PMC3933005

[23] Rau C. D., Wang J., Avetisyan R., Romay M. C., Martin L., Ren S., Wang Y., and Lusis A. J. 2015 Mapping genetic contributions to cardiac pathology induced by Beta-adrenergic stimulation in mice. Circ Cardiovasc Genet. 8: 40–49. 25480693 10.1161/CIRCGENETICS.113.000732 PMC4334708

[24] Hasin-Brumshtein Y., Hormozdiari F., Martin L., van Nas A., Eskin E., Lusis A. J., and Drake T. A. 2014 Allele-specific expression and eQTL analysis in mouse adipose tissue. BMC Genomics. 15: 471. 24927774 10.1186/1471-2164-15-471 PMC4089026

[25] Lagarrigue S., Martin L., Hormozdiari F., Roux P. F., Pan C., van Nas A., Demeure O., Cantor R., Ghazalpour A., Eskin E., 2013 Analysis of allele-specific expression in mouse liver by RNA-Seq: a comparison with Cis-eQTL identified using genetic linkage. Genetics. 195: 1157–1166. 24026101 10.1534/genetics.113.153882 PMC3813844

[26] Civelek M., and Lusis A. J. 2014 Systems genetics approaches to understand complex traits. Nat. Rev. Genet. 15: 34–48. 24296534 10.1038/nrg3575 PMC3934510

[27] Machleder D., Ivandic B., Welch C., Castellani L., Reue K., and Lusis A. J. 1997 Complex genetic control of HDL levels in mice in response to an atherogenic diet. Coordinate regulation of HDL levels and bile acid metabolism. J. Clin. Invest. 99: 1406–1419. 9077551 10.1172/JCI119300 PMC507957

[28] Montgomery S. B., and Dermitzakis E. T. 2011 From expression QTLs to personalized transcriptomics. Nat. Rev. Genet. 12: 277–282. 21386863 10.1038/nrg2969

[29] Brænne I., Civelek M., Vilne B., Di Narzo A., Johnson A. D., Zhao Y., Reiz B., Codoni V., Webb T. R., Foroughi Asl H., ; Leducq Consortium CAD Genomics. 2015 Prediction of causal candidate genes in coronary artery disease loci. Arterioscler. Thromb. Vasc. Biol. 35: 2207–2217. 26293461 10.1161/ATVBAHA.115.306108 PMC4583353

[30] Schadt E. E., Lamb J., Yang X., Zhu J., Edwards S., Guhathakurta D., Sieberts S. K., Monks S., Reitman M., Zhang C., 2005 An integrative genomics approach to infer causal associations between gene expression and disease. Nat. Genet. 37: 710–717. 15965475 10.1038/ng1589 PMC2841396

[31] Aten J. E., Fuller T. F., Lusis A. J., and Horvath S. 2008 Using genetic markers to orient the edges in quantitative trait networks: the NEO software. BMC Syst. Biol. 2: 34. 18412962 10.1186/1752-0509-2-34 PMC2387136

[32] Lan H., Chen M., Flowers J. B., Yandell B. S., Stapleton D. S., Mata C. M., Mui E. T., Flowers M. T., Schueler K. L., Manly K. F., 2006 Combined expression trait correlations and expression quantitative trait locus mapping. PLoS Genet. 2: e6. 16424919 10.1371/journal.pgen.0020006 PMC1331977

[33] Farber C. R. 2013 Systems-level analysis of genome-wide association data. G3 (Bethesda). 3: 119–129. 23316444 10.1534/g3.112.004788 PMC3538337

[34] Rau C. D., Wisniewski N., Orozco L. D., Bennett B., Weiss J., and Lusis A. J. 2013 Maximal information component analysis: a novel non-linear network analysis method. Front. Genet. 4: 28. 23487572 10.3389/fgene.2013.00028 PMC3594742

[35] Kebede M. A., and Attie A. D. 2014 Insights into obesity and diabetes at the intersection of mouse and human genetics. Trends Endocrinol. Metab. 25: 493–501. 25034129 10.1016/j.tem.2014.06.006 PMC4177963

[36] Izawa T., Rohatgi N., Fukunaga T., Wang Q. T., Silva M. J., Gardner M. J., McDaniel M. L., Abumrad N. A., Semenkovich C. F., Teitelbaum S. L., 2015 ASXL2 regulates glucose, lipid, and skeletal homeostasis. Cell Reports. 11: 1625–1637. 26051940 10.1016/j.celrep.2015.05.019 PMC4472564

[37] Langfelder P., and Horvath S. 2008 WGCNA: an R package for weighted correlation network analysis. BMC Bioinformatics. 9: 559. 19114008 10.1186/1471-2105-9-559 PMC2631488

[38] Calabrese G., Bennett B. J., Orozco L., Kang H. M., Eskin E., Dombret C., De Backer O., Lusis A. J., and Farber C. R. 2012 Systems genetic analysis of osteoblast-lineage cells. PLoS Genet. 8: e1003150. 23300464 10.1371/journal.pgen.1003150 PMC3531492

[39] Meyre D., Delplanque J., Chevre J. C., Lecoeur C., Lobbens S., Gallina S., Durand E., Vatin V., Degraeve F., Proenca C., 2009 Genome-wide association study for early-onset and morbid adult obesity identifies three new risk loci in European populations. Nat. Genet. 41: 157–159. 19151714 10.1038/ng.301

[40] Locke A. E., Kahali B., Berndt S. I., Justice A. E., Pers T. H., Day F. R., Powell C., Vedantam S., Buchkovich M. L., Yang J., 2015 Genetic studies of body mass index yield new insights for obesity biology. Nature. 518: 197–206. 25673413 10.1038/nature14177 PMC4382211

[41] Jelinek D., Heidenreich R. A., and Garver W. S. 2011 The Niemann-Pick C1 gene interacts with a high-fat diet and modifying genes to promote weight gain. Am. J. Med. Genet. A. 155A: 2317–2319. 21815257 10.1002/ajmg.a.34135

[42] Org E., Parks B. W., Joo J. W., Emert B., Schwartzman W., Kang E. Y., Mehrabian M., Pan C., Knight R., Gunsalus R., 2015 Genetic and environmental control of host-gut microbiota interactions. Genome Res. 25: 1558–1569. 26260972 10.1101/gr.194118.115 PMC4579341

[43] Cox L. M., and Blaser M. J. 2015 Antibiotics in early life and obesity. Nat. Rev. Endocrinol. 11: 182–190. 25488483 10.1038/nrendo.2014.210 PMC4487629

[44] Hui S. T., Parks B. W., Org E., Norheim F., Che N., Pan C., Castellani L. W., Charugundla S., Dirks D. L., Psychogios N., 2015 The genetic architecture of NAFLD among inbred strains of mice. eLife. 4: e05607. 26067236 10.7554/eLife.05607 PMC4493743

[45] Morrison A. C., Felix J. F., Cupples L. A., Glazer N. L., Loehr L. R., Dehghan A., Demissie S., Bis J. C., Rosamond W. D., Aulchenko Y. S., 2010 Genomic variation associated with mortality among adults of European and African ancestry with heart failure: the cohorts for heart and aging research in genomic epidemiology consortium. Circ Cardiovasc Genet. 3: 248–255. 20400778 10.1161/CIRCGENETICS.109.895995 PMC3033765

[46] Villard E., Perret C., Gary F., Proust C., Dilanian G., Hengstenberg C., Ruppert V., Arbustini E., Wichter T., Germain M., 2011 A genome-wide association study identifies two loci associated with heart failure due to dilated cardiomyopathy. Eur. Heart J. 32: 1065–1076. 21459883 10.1093/eurheartj/ehr105 PMC3086901

[47] Meng H., Vera I., Che N., Wang X., Wang S. S., Ingram-Drake L., Schadt E. E., Drake T. A., and Lusis A. J. 2007 Identification of Abcc6 as the major causal gene for dystrophic cardiac calcification in mice through integrative genomics. Proc. Natl. Acad. Sci. USA. 104: 4530–4535. 17360558 10.1073/pnas.0607620104 PMC1838635

[48] LeBoeuf R. C., Puppione D. L., Schumaker V. N., and Lusis A. J. 1983 Genetic control of lipid transport in mice. I. Structural properties and polymorphisms of plasma lipoproteins. J. Biol. Chem. 258: 5063–5070. 6833293

[49] Bennett B. J., Davis R. C., Civelek M., Orozco L., Wu J., Qi H., Pan C., Packard R. R. S., Eskin E., Yan M., 2015 Genetic architecture of atherosclerosis in mice: a systems genetics analysis of common inbred strains. PLoS Genet. 11: e1005711. 26694027 10.1371/journal.pgen.1005711 PMC4687930

[50] Talukdar H. A., Foroughi Asl H., Jain R., Ermel R., Ruusalepp A., Franze O., Kidd B. A., Readhead B., Giannarelli C., Kovacic J. C., 2016 Cross-tissue regulatory gene networks in coronary artery disease. Cell Syst. 2: 196–208. 27135365 10.1016/j.cels.2016.02.002 PMC4855300

[51] van Nas A., Ingram-Drake L., Sinsheimer J. S., Wang S. S., Schadt E. E., Drake T., and Lusis A. J. 2010 Expression quantitative trait loci: replication, tissue- and sex-specificity in mice. Genetics. 185: 1059–1068. 20439777 10.1534/genetics.110.116087 PMC2907192

[52] Hiyari S., Atti E., Camargo P. M., Eskin E., Lusis A. J., Tetradis S., and Pirih F. Q. 2015 Heritability of periodontal bone loss in mice. J. Periodontal Res. 50: 730–736. 25581386 10.1111/jre.12258 PMC4499504

[53] Wu X., Davis R. C., McMillen T. S., Schaeffer V., Zhou Z., Qi H., Mazandarani P. N., Alialy R., Hudkins K. L., Lusis A. J., 2014 Genetic modulation of diabetic nephropathy among mouse strains with Ins2 Akita mutation. Physiol. Rep. 2: e12208. 25428948 10.14814/phy2.12208 PMC4255814

[54] Crow A. L., Ohmen J., Wang J., Lavinsky J., Hartiala J., Li Q., Li X., Salehide P., Eskin E., Pan C., 2015 The genetic architecture of hearing impairment in mice: evidence for frequency-specific genetic determinants. G3 (Bethesda). 5: 2329–2339. 26342000 10.1534/g3.115.021592 PMC4632053

[55] Lavinsky J., Crow A. L., Pan C., Wang J., Aaron K. A., Ho M. K., Li Q., Salehide P., Myint A., Monges-Hernadez M., 2015 Correction: genome-wide association study identifies Nox3 as a critical gene for susceptibility to noise-induced hearing loss. PLoS Genet. 11: e1005293. 25880434 10.1371/journal.pgen.1005094 PMC4399881

[56] Knight J. C. 2014 Approaches for establishing the function of regulatory genetic variants involved in disease. Genome Med. 6: 92. 25473428 10.1186/s13073-014-0092-4 PMC4254439

[57] Shao H., Burrage L. C., Sinasac D. S., Hill A. E., Ernest S. R., O’Brien W., Courtland H. W., Jepsen K. J., Kirby A., Kulbokas E. J., 2008 Genetic architecture of complex traits: large phenotypic effects and pervasive epistasis. Proc. Natl. Acad. Sci. USA. 105: 19910–19914. 19066216 10.1073/pnas.0810388105 PMC2604967

[58] Tian J., Keller M. P., Oler A. T., Rabaglia M. E., Schueler K. L., Stapleton D. S., Broman A. T., Zhao W., Kendziorski C., Yandell B. S., 2015 Identification of the bile acid transporter Slco1a6 as a candidate gene that broadly affects gene expression in mouse pancreatic islets. Genetics. 201: 1253–1262. 26385979 10.1534/genetics.115.179432 PMC4649649

[59] Albert F. W., Treusch S., Shockley A. H., Bloom J. S., and Kruglyak L. 2014 Genetics of single-cell protein abundance variation in large yeast populations. Nature. 506: 494–497. 24402228 10.1038/nature12904 PMC4285441

[60] Shen Y., Yue F., McCleary D. F., Ye Z., Edsall L., Kuan S., Wagner U., Dixon J., Lee L., Lobanenkov V. V., 2012 A map of the cis-regulatory sequences in the mouse genome. Nature. 488: 116–120. 22763441 10.1038/nature11243 PMC4041622

[61] ENCODE Project Consortium. 2012 An integrated encyclopedia of DNA elements in the human genome. Nature. 489: 57–74. 22955616 10.1038/nature11247 PMC3439153

[62] Leung A., Parks B. W., Du J., Trac C., Setten R., Chen Y., Brown K., Lusis A. J., Natarajan R., and Schones D. E. 2014 Open chromatin profiling in mice livers reveals unique chromatin variations induced by high fat diet. J. Biol. Chem. 289: 23557–23567. 25006255 10.1074/jbc.M114.581439 PMC4156056

[63] Orozco L. D., Morselli M., Rubbi L., Guo W., Go J., Shi H., Lopez D., Furlotte N. A., Bennett B. J., Farber C. R., 2015 Epigenome-wide association of liver methylation patterns and complex metabolic traits in mice. Cell Metab. 21: 905–917. 26039453 10.1016/j.cmet.2015.04.025 PMC4454894

[64] Orozco L. D., Rubbi L., Martin L. J., Fang F., Hormozdiari F., Che N., Smith A. D., Lusis A. J., and Pellegrini M. 2014 Intergenerational genomic DNA methylation patterns in mouse hybrid strains. Genome Biol. 15: R68. 24887417 10.1186/gb-2014-15-5-r68 PMC4076608

[65] Nicholson G., Rantalainen M., Maher A. D., Li J. V., Malmodin D., Ahmadi K. R., Faber J. H., Hallgrimsdottir I. B., Barrett A., Toft H., 2011 Human metabolic profiles are stably controlled by genetic and environmental variation. Mol. Syst. Biol. 7: 525. 21878913 10.1038/msb.2011.57 PMC3202796

[66] Org E., Mehrabian M., and Lusis A. J. 2015 Unraveling the environmental and genetic interactions in atherosclerosis: central role of the gut microbiota. Atherosclerosis. 241: 387–399. 26071662 10.1016/j.atherosclerosis.2015.05.035 PMC4510029

[67] Bennett B. J., de Aguiar Vallim T. Q., Wang Z., Shih D. M., Meng Y., Gregory J., Allayee H., Lee R., Graham M., Crooke R., 2013 Trimethylamine-N-oxide, a metabolite associated with atherosclerosis, exhibits complex genetic and dietary regulation. Cell Metab. 17: 49–60. 23312283 10.1016/j.cmet.2012.12.011 PMC3771112

[68] Gregory J. C., Buffa J. A., Org E., Wang Z., Levison B. S., Zhu W., Wagner M. A., Bennett B. J., Li L., DiDonato J. A., 2015 Transmission of atherosclerosis susceptibility with gut microbial transplantation. J. Biol. Chem. 290: 5647–5660. 25550161 10.1074/jbc.M114.618249 PMC4342477

[69] Goodrich J. K., Waters J. L., Poole A. C., Sutter J. L., Koren O., Blekhman R., Beaumont M., Van Treuren W., Knight R., Bell J. T., 2014 Human genetics shape the gut microbiome. Cell. 159: 789–799. 25417156 10.1016/j.cell.2014.09.053 PMC4255478

[70] Arnold A. P., and Lusis A. J. 2012 Understanding the sexome: measuring and reporting sex differences in gene systems. Endocrinology. 153: 2551–2555. 22434084 10.1210/en.2011-2134 PMC3359607

[71] Yang X., Schadt E. E., Wang S., Wang H., Arnold A. P., Ingram-Drake L., Drake T. A., and Lusis A. J. 2006 Tissue-specific expression and regulation of sexually dimorphic genes in mice. Genome Res. 16: 995–1004. 16825664 10.1101/gr.5217506 PMC1524872

[72] van Nas A., Guhathakurta D., Wang S. S., Yehya N., Horvath S., Zhang B., Ingram-Drake L., Chaudhuri G., Schadt E. E., Drake T. A., 2009 Elucidating the role of gonadal hormones in sexually dimorphic gene coexpression networks. Endocrinology. 150: 1235–1249. 18974276 10.1210/en.2008-0563 PMC2654741

[73] van der Harst P., Zhang W., Mateo Leach I., Rendon A., Verweij N., Sehmi J., Paul D. S., Elling U., Allayee H., Li X., 2012 Seventy-five genetic loci influencing the human red blood cell. Nature. 492: 369–375. 23222517 10.1038/nature11677 PMC3623669

[74] Zhou X., Crow A. L., Hartiala J., Spindler T. J., Ghazalpour A., Barsky L. W., Bennett B. J., Parks B. W., Eskin E., Jain R., 2015 The genetic landscape of hematopoietic stem cell frequency in mice. Stem Cell Reports. 5: 125–138. 26050929 10.1016/j.stemcr.2015.05.008 PMC4618249

[75] van Nas A., Pan C., Ingram-Drake L. A., Ghazalpour A., Drake T. A., Sobel E. M., Papp J. C., and Lusis A. J. 2013 The systems genetics resource: a web application to mine global data for complex disease traits. Front. Genet. 4: 84. 23730305 10.3389/fgene.2013.00084 PMC3657633

[76] Ghazalpour A., Doss S., Zhang B., Wang S., Plaisier C., Castellanos R., Brozell A., Schadt E. E., Drake T. A., Lusis A. J., 2006 Integrating genetic and network analysis to characterize genes related to mouse weight. PLoS Genet. 2: e130. 16934000 10.1371/journal.pgen.0020130 PMC1550283

[77] Buchner D. A., and Nadeau J. H. 2015 Contrasting genetic architectures in different mouse reference populations used for studying complex traits. Genome Res. 25: 775–791. 25953951 10.1101/gr.187450.114 PMC4448675

